# Glucocorticoid Receptor Signaling: Multilevel Organization, Roles in Fetal Development, and Postnatal Outcomes

**DOI:** 10.3390/ijms27062873

**Published:** 2026-03-22

**Authors:** Sofiya Potapova, Yan Isakov, Ekaterina Tyulkova, Oleg Vetrovoy

**Affiliations:** Laboratory of Regulation of Brain Neuronal Functions, Pavlov Institute of Physiology, Russian Academy of Sciences, Makarova Emb. 6, 199034 Saint-Petersburg, Russia

**Keywords:** hypothalamic–pituitary–adrenal axis, glucocorticoid receptor, maternal stress, fetal development, postnatal outcomes

## Abstract

The hypothalamic–pituitary–adrenal (HPA) axis coordinates metabolic, immune, and behavioral responses to a changing environment. Its molecular effectors are the nuclear receptors for glucocorticoids and mineralocorticoids (the GRs/MRs), encoded by *nr3c1*/*nr3c2*. The MR serves as the high-affinity sensor of basal hormone concentrations, whereas the GR amplifies the stress response and mediates negative feedback. Despite their shared domain architecture, the receptors have diverged functionally: isoform composition, post-translational modifications, and the complement of co-regulators together determine which genes are activated or repressed in a given tissue at a given time. The regulation of the HPA axis activity is a major determinant of embryonic development. Pregnancy adds a placental control layer that meters maternal signals: 11β-hydroxysteroid dehydrogenase type 2 (11β-HSD2) in the syncytiotrophoblast inactivates cortisol, whereas 11β-hydroxysteroid dehydrogenase type 1 (11β-HSD1) can regenerate it, and systemic buffering by transcortin (cortisol-binding globulin, CBG) limits the free hormone fraction. Under stress, inflammation, or hypoxia, this barrier weakens, exposing the fetus to stronger glucocorticoid pulses during windows of heightened vulnerability for brain and immune development. Such overexposure not only reshapes ongoing transcription but is also epigenetically inscribed: the methylation of alternative *nr3c1* promoters, the remodeling of histones, and the shifts in ncRNA profiles recalibrate the axis sensitivity for the long term. At the phenotypic level, this manifests as variability in stress reactivity, cognitive and affective trajectories, and an immune and metabolic risk across later ontogeny. In this review, we integrate evidence on the structure and functions of the GR, the mechanisms of its post-translational and epigenetic regulation, and the role of the placenta, to provide a coherent framework for understanding the multifaceted consequences of prenatal stress and to identify potential targets for early prevention.

## 1. Introduction

A robust adaptation to environmental change from metabolic shifts to immune challenges and behavioral responses is orchestrated by the hypothalamic–pituitary–adrenal (HPA) axis, whose principal effectors are the glucocorticoid and mineralocorticoid receptors (the GR and MR) [1,2,3,4,5,6,7,8,9]. These closely related nuclear receptors share a common domain architecture yet have functionally diverged: they exhibit differences in their isoform composition, their post-translational modifications, and their co-regulator availability to confer tissue-specific transcriptional outcomes. During pregnancy, an additional regulatory layer at the maternal–fetal interface comes into play: the placenta is not a passive conduit but an active gatekeeper of glucocorticoid signals. The enzyme pair 11β-hydroxysteroid dehydrogenase type 2 (11β-HSD2) and type 1 (11β-HSD1) [10,11,12,13,14], together with corticosteroid-binding globulin (CBG), buffer the fetus from maternal cortisol surges by inactivating excess cortisol and limiting the free hormone fraction [15,16]. However, maternal stress, inflammation, or hypoxia can compromise this placental barrier [17,18,19]. As a result, louder and longer glucocorticoid pulses reach the fetus precisely during critical windows of tissue development. Such prenatal glucocorticoid overexposure not only perturbs the ongoing gene expression but is also epigenetically inscribed into developing cells, ultimately recalibrating the offspring’s physiological set-points. In addition to the well-documented impacts on neurodevelopment and immune maturation, this fetal programming extends to the reproductive endocrine axis: prenatal stress can disrupt the hypothalamic–pituitary–gonadal (HPG) axis, altering the gonadotropin release and the gonadal development in a sex-dependent manner [20,21]. The fine-tuning of the HPA axis activity in utero is thus a major determinant of the offspring’s developmental trajectory. The maternal stress response shapes not only the mother’s well-being but also the long-term “setup” of the child’s neuroendocrine, immune, metabolic, and reproductive systems. Emerging evidence even suggests transgenerational consequences of prenatal stress, with epigenetic alterations detectable in the sperm and, to a lesser extent, the oocytes of the exposed offspring [22,23,24,25,26,27]. In summary, glucocorticoid regulation in pregnancy is pivotal for reproductive biology, as the mother–placenta–fetus unit orchestrates fetal development and can imprint changes that persist throughout the offspring’s life and potentially into subsequent generations.

The classical view of glucocorticoids as simple on/off triggers of gene transcription is insufficient to explain the nuanced, tissue-specific outcomes of prenatal stress. The GR functionality is specified by multi-layered regulatory machinery: the genomic organization of *nr3c1*/*nr3c2* and an array of alternative first exons drive cell-type-specific expression [28]; chaperone complexes (Hsp90/Hsp70 with co-chaperones FKBP51/52) control ligand-binding kinetics and nuclear transport; post-translational modifications (phosphorylation, acetylation, SUMOylation, ubiquitination) steer the balance between the GR’s transactivating and transrepressing actions [29,30,31,32,33,34,35,36,37,38,39,40,41,42]; and local ligand availability is modulated by tissue-specific 11β-HSD enzymes [10,11,12,13,14]. Acting in concert, these mechanisms explain how an identical hormonal signal can yield opposite effects in different contexts. The epigenetic layer of regulation is equally critical. The prenatal cortisol spikes leave a molecular “memory” in the fetal genome: DNA methylation changes at key regulatory loci (for instance, the GR (*nr3c1*) promoter variants and enhancers of the GR-responsive genes), remodeling of histone marks, and shifts in noncoding RNA (miRNA) networks can durably re-tune the HPA axis activity [43,44]. This stress-induced reprogramming often weakens the HPA negative feedback and skews the balance of the GR/MR signaling, thereby stabilizing a phenotype of heightened stress reactivity and altered immune function. The magnitude and direction of these effects are modulated by the timing of exposure (gestational window), the circadian context of maternal–fetal hormone rhythms, and fetal sex.

This review assembles an unbroken causal chain from maternal stress triggers to offspring outcomes through the lens of the GR-centric mechanisms. We begin by examining the structure and genomic organization of GRs and MRs, highlighting features that underline their tissue-specific actions. We then analyze the dynamic regulatory mechanisms that modulate the GR signaling: chaperone-mediated assembly, key post-translational modifications, and their functional consequences. A dedicated section addresses the epigenetic regulation of *nr3c1* and illustrates how prenatal adverse conditions become encoded in chromatin. Next, we explore how the placenta doses or dampens the maternal signals (via 11β-HSD2/11β-HSD1 activity, CBG, and the transport of exogenous glucocorticoids), and we explore the circumstances under which this maternal–fetal barrier fails, linking placental dysfunction with maternal endocrine stress responses (CRH–ACTH–cortisol feedback loops, uteroplacental blood flow) and tracing the impact it has on the fetal GR programming. Finally, we synthesize evidence for the long-term outcomes in the offspring, including affective and stress-related disorders, cardiometabolic risk (insulin resistance, hypertension), immune/inflammatory dysregulation (e.g., relative glucocorticoid resistance), disturbances of the reproductive axis, and even increased vulnerability to substance misuse. Our aim is to provide a coherent mechanistic framework that explains how transient prenatal stress exposures are consolidated into lasting developmental “recalibrations” and to identify the most promising leverage points for preventive or early therapeutic interventions in the context of reproductive health.

## 2. Structure of the GR and the MR

### 2.1. Comparison of nr3c1 and nr3c2 Genes Organization

The glucocorticoid (GR) and mineralocorticoid (MR) receptors belong to the nuclear receptor family of steroid hormone receptors and are encoded by *nr3c1* and *nr3c2*, respectively. The two genes share a broadly similar modular architecture, reflecting that they have descended from a common ancestor by duplication, yet they differ in the extent of their regulatory landscapes and in the complexity of the 5′ untranslated region (5′-UTR).

The *nr3c1* gene is located on human chromosome 5 [45,46]. It comprises nine exons: the first encodes the 5′ untranslated region, while exons 2–9 encode the GR protein. Although the 5′UTR does not encode a polypeptide and does not alter downstream exon composition (exon 2 has a single universal acceptor site), it regulates translation and thus determines the cellular receptor abundance. Up to 14 first-exon variants have been described [47]; 1G remains putative (predicted by homology to the rat exon 1–8), and 1I and 1J are rare in humans but have been confirmed in pigs. The first exons are organized in two promoter regions: 1A (1–3) and 1I in a distal domain, with the remaining first exons in a proximal domain. Exon 9 contains two alternative splice acceptor sites, yielding the α and β variants.

*nr3c2* resides on chromosome 4q31 [48,49,50] and is more expansive than *nr3c1*: its genomic region contains nine exons and features a considerably shorter and less diverse 5′-UTR that is represented by only two specific variants [51] and is likewise encoded by the first exon. As in *nr3c1*, the universal exon 2 initiates the coding of the N-terminal domain, followed by exons that form the DNA-binding domain, the hinge region, and the ligand-binding domain.

**Figure 1:**
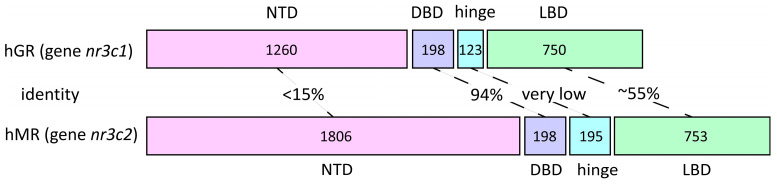
The GR and MR homology. The schematic domain architecture of the human glucocorticoid receptor (GR/*nr3c1*) and the mineralocorticoid receptor (MR/*nr3c2*). The boxes show the four canonical regions—the N-terminal domain with AF-1 (NTD/AF-1), the DNA-binding domain (DBD; two zinc fingers), the hinge, and the ligand-binding domain with AF-2 (LBD/AF-2). The numbers inside the boxes indicate the coding length in nucleotides for each domain (the GR: 1260/198/123/750; the MR: 1806/198/195/753), corresponding to ~420/66/41/250 and ~602/66/65/251 amino acids, respectively.

Beyond the substantial differences in the intron length and the 5′UTR, the overall architecture of the two genes is quite similar (Figure 1) and is thought to reflect duplication from a common ancestor. Comparable, though not identical, organizations are observed in other members of the family, such as *nr3c3* and *nr3c4* [51], which encode the progesterone and androgen receptors, respectively, which are consistent with their evolutionary relatedness and the similarity of their ligands. The evidence for shared ancestry comes not only from this common organizational logic but also from a high sequence correspondence; for example, in rats, *nr3c1* and *nr3c2* exhibit ~76% [52] nucleotide identity within the DBD. Further details on the receptor homology are provided below.

### 2.2. GR and MR Proteins: Degree of Homology and Functional Divergence

The overall structure of GR and MR protein domains [28] is similar, but the extent of conservation differs across domains.

The MR and GR exhibit a broadly comparable affinity for cortisol (Kd ~ 1–5 nM), corticosterone (Kd ~ 3–5 nM), and dexamethasone (Kd ~ 0.5–3 nM), but the MR additionally recognizes aldosterone with high affinity [53,54], whereas the GR does not [47]. The shared specificity for cortisol and corticosterone reflects conservation across key LBD motifs. At the same time, the MR’s ability—unlike GR’s—to bind aldosterone with high affinity, which maps to residues 820–844 [55] (as shown using MR/GR chimeras), is intriguingly positioned on the receptor’s surface rather than in the ligand-binding pocket. The ligand engagement of the pocket is necessary for co-regulator recruitment, which occurs through the AF-2 region of the LBD. A much greater similarity between the two receptors is observed in the DBD (~94% identity). The DBD mediates DNA binding (via two zinc-finger motifs) and dimerization, and it also harbors a nuclear localization signal [56]. By contrast, the NTD of nuclear receptors is the least conserved region (<15% amino-acid identity between the MR and the GR). Unlike the LBD and DBD, the NTD has a poorly ordered secondary structure, which confers the conformational flexibility needed to engage diverse coregulators by adopting multiple stable secondary structures within the activation function-1 (AF-1) region [57,58,59,60,61]. A further key feature of the NTD is that it contains numerous sites for post-translational modification.

Domain homology tracks with a division of physiological labor. Functionally, the GR is the principal driver of metabolic and stress adaptation: it induces gluconeogenic programs (PCK1, G6PC, TAT, FBP1) [62,63,64,65], rewires signaling kinases [66] and transcription factors [67], suppresses pro-inflammatory cascades [68], and mediates negative feedback within the HPA axis [1,2]. The MR is the sensor for water–salt balance and osmotic tone, governing the expression of ENaC [69,70], Na^+^/K^+^-ATPase [71], and their regulators [72,73]; it also contributes to cellular proliferation/survival and, depending on the context, can potentiate inflammatory responses [74,75,76]. The MR participates in additional processes, including protecting cells from apoptosis [77,78,79] (including that induced by GR) and the mediation of stress responses via rapid non-genomic signaling [80,81,82,83].

The ability of structurally similar ligands to act on the receptors of similar architectures yet elicit markedly different physiological effects—and to do so in distinct tissues—has long remained puzzling [84]; even now, not all facets of the GR/MR specificity are fully understood. The core difficulty is twofold. First, as noted above, the DBD sequences of the two receptor types are highly homologous, meaning they essentially recognize the same DNA motifs [85]. Second, the MR binds glucocorticoids with sufficient affinity so that, in principle, the MR would be tonically activated by corticosterone/cortisol even at basal concentrations (as observed in vitro) were it not for additional regulatory mechanisms that operate in vivo. Such a divergence of effects, despite a shared “nuclear toolkit” in the DBD, is a direct product of domain-level differences, chromatin context, and co-factor landscapes.

Accordingly, the glucocorticoid entry into tissues—and their transcriptional output—is regulated at multiple levels. Most of the circulating glucocorticoids are bound to CBG, unlike aldosterone; only the free hormone can enter cells, thereby lowering the concentration available for receptor activation. In classical mineralocorticoid-sensitive epithelia [86,87] (kidney, parotid gland, and distal colon), the principal barrier to glucocorticoids is 11β-hydroxysteroid dehydrogenase type 2 (11β-HSD2) [88,89], which converts cortisol and corticosterone—but not aldosterone—into their inert 11-keto analogs that cannot activate MRs, effectively rendering the MR in these tissues aldosterone-selective. The first isoenzyme, 11β-HSD1 [10], possesses both 11β-dehydrogenase and 11β-reductase activities (predominantly the latter in vivo) and was first purified from the liver [11,12,13]. Its physiological role is not fully resolved; in most cells, it primarily converts cortisone to cortisol, increasing the pool of active glucocorticoids [14]. In tissues lacking 11β-HSD2, both the MR and the GR are activated by glucocorticoids and would, at first glance, be expected to act on the same target genes.

Thus, despite the high overall homology of the GR and the MR, their tissue specificity and physiological effects are shaped by a composite of factors, including differences in ligand affinity, local glucocorticoid inactivation, and interactions with co-regulatory proteins [55,88,90,91]. In the remainder of this review, we focus on GRs as the key mediators of prenatal-stress effects and the central players in fetal programming.

### 2.3. The GR Isoforms: α, β, γ, Rare Splice Variants, and N-Terminal Translational Isoforms

By “canonical GR” one typically refers to GRα, produced by splicing at the proximal acceptor site in exon 9 and encoding a 777-aa protein. This isoform binds natural and synthetic glucocorticoids and dimerizes, translocates to the nucleus, and recruits co-activator complexes via AF-2.

Alternative splicing of the same exon 9 yields GRβ [46], which lacks a properly formed ligand-binding pocket due to the replacement of the C-terminal segment; consequently, endogenous glucocorticoids do not activate it. Thus, GRβ cannot bind corticosteroids and remains an “orphan” receptor, and its physiological ligand, if any, is unknown [92,93]. Functionally, GRβ exerts dominant negative effects on GRα: when GRβ exceeds GRα, transcriptional activation or repression normally driven by GRα is attenuated [94]. The proposed mechanisms include the competitive binding of GRβ homodimers to glucocorticoid response elements (GREs) and the formation of transcriptionally inactive GRα/GRβ heterodimers that effectively “switch off” GRα. In addition, GRβ has GRα-independent transcriptional activity that largely targets glucocorticoid-independent genes [95,96]. Although overexpression of GRβ is frequently associated with pathophysiology and glucocorticoid resistance [97,98,99], GRβ is the predominant isoform in some normal tissues and participates in physiological processes [100,101,102].

A further stable splice variant is GRγ, which differs by the insertion of a single arginine [103] between the two zinc fingers of the DBD owing to alternative splice-site usage. This seemingly minimal change shifts nuclear–cytoplasmic distribution (GRγ is more often retained in the cytoplasm, making it a candidate for the still elusive membrane GRs) [104]. GRγ binds GREs with an affinity comparable to GRα, and the two share many targets, yet their specificities are not identical, with genes regulated by one but not the other [105]. The differences in specificity and localization are attributable to the arginine insertion near the NL1 nuclear localization sequence within the DBD. Its physiological function is not fully defined; nevertheless, GRγ has been implicated in the regulation of cellular energy metabolism [104].

The less common C-terminally truncated (GR-A and GR-P) variants have also been described. GR-P lacks exon 8 [106] and all downstream exons at the mRNA level: the LBD C-terminus is shortened, and both the dimerization signal and the AF-2 are absent, while a fragment of intron 7 is retained, rendering the C-terminus unique. The resulting 647-aa protein cannot bind to a ligand. GR-P is expressed in both normal and malignant cells; in some contexts, its expression is associated with glucocorticoid resistance [107], whereas in others it paradoxically enhances glucocorticoid-dependent signaling [108].

Similarly, GR-A arises from aberrant splicing: the donor site of exon 4 joins the acceptor of exon 8, thereby omitting exons 5–7 [106], which encode part of the LBD, including AF-2 and a nuclear localization signal. GR-A was first identified in myeloma cells with a distinctive expression pattern: it is absent in glucocorticoid-sensitive cells, present at early stages of resistance, and absent again once resistance is fully established [107].

Beyond splice-derived isoforms, translational initiation at alternative AUG codons within the N-terminal domain (NTD) generates a set of N-terminally truncated translational isoforms. The GR mRNA contains eight AUGs, each potentially initiating translation, yielding eight NTD variants that differ only in length, all of which have been confirmed for GRα (analogous truncated NTDs for other isoforms remain possible). Because the LBD remains intact, these isoforms retain the ligand binding. Although the DBD is also intact, they nonetheless occupy distinct genomic targets.

The variants summarized here (Table 1)—along with additional diversity arising from deletions, insertions, and SNPs—collectively turn GR into a family of proteins rather than a single gene product [109,110,111,112,113]. This seemingly semantic point has direct consequences for experimental interpretation, for the clinical landscape of glucocorticoid resistance, and for understanding which epigenetic mechanisms govern each isoform’s contribution to tissue-specific stress responses, which is central to this review.

In the placenta, this isoform diversity provides a critical buffer against maternal physiological stressors; however, prenatal stress can pathologically “reprogram” this balance. For instance, recent studies have demonstrated that maternal anxiety and depression are associated with an upregulation of the GRα-D1 isoform, correlating with a pro-inflammatory placental environment [119]. Furthermore, exposure to synthetic glucocorticoids has been shown to increase pro-apoptotic isoforms like GRαC and GR-P in a sex-specific manner, particularly in female fetuses [120]. This shift towards less transcriptionally active variants can fundamentally alter the placental response to circulating cortisol, often resulting in tissue-specific glucocorticoid resistance and the disinhibition of pro-inflammatory cytokine cascades [121]. Consequently, such molecular alterations are linked to a spectrum of adverse outcomes, including impaired trophoblast invasion, reduced fetoplacental vascularization, and a compromised barrier function, ultimately contributing to intrauterine growth restriction (IUGR) and the fetal programming of lifelong disease [121,122]. Of course, there is not enough data on the time- and context-dependent composition of the GR isoforms, but the data available in the literature, as well as our understanding of the functions of different isoforms, suggest that this is an important mechanism for regulating glucocorticoid signaling in the developing fetus.

### 2.4. Nuclear Translocation and Chaperone Complexes

**Figure 2:**
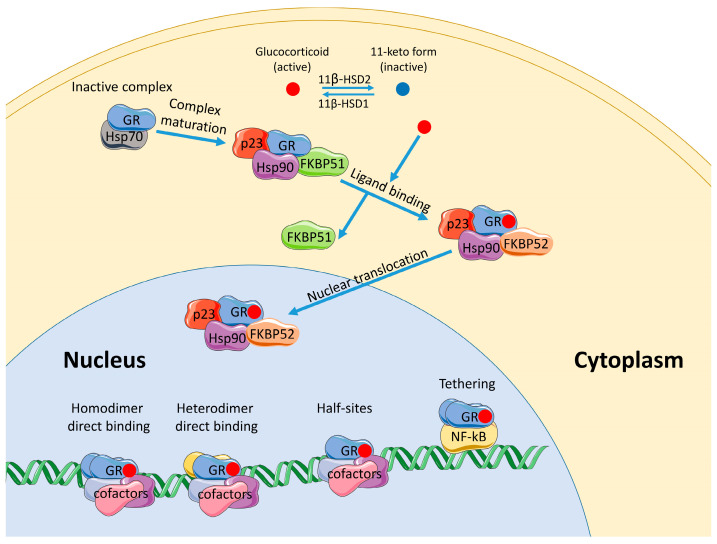
The chaperone-guided activation, nuclear translocation, and DNA engagement modes of the GR. The cytosolic GR cycles through an Hsp70 → Hsp90 maturation pathway. In the client-loading state, GR associates with Hsp90 and p23 and, in the presence of FKBP51, remains in a low-affinity, cytoplasmic complex. Ligand binding (active glucocorticoid; availability shaped by interconversion via 11β-HSD2 → inactive 11-keto and 11β-HSD1 → active cortisol) promotes complex tightening and a functional switch from FKBP51 to FKBP52, which facilitates the nuclear import. In the nucleus, the GR engages chromatin in multiple modes: homodimeric binding at canonical GREs to activate or repress target genes; heterodimeric interactions with related steroid receptors on composite elements; recognition of half-sites/cryptic GREs; and tethered transrepression, where the GR suppresses inflammatory programs by a protein–protein interaction with NF-κB. The recruitment of co-regulators at each mode determines the balance between transactivation and transrepression. The figure was created using Servier Medical Art (https://smart.servier.com/), licensed under CC BY 4.0 (https://creativecommons.org/licenses/by/4.0/ (accessed on 10 August 2025)).

At rest, the GR continuously shuttles between the cytoplasm and the nucleus [29,30,31]. This dynamic equilibrium is set by the balance of the nuclear import and export signals within the receptor itself (NLS/NES) and by the state of its protein milieu [32,33]. The engagement of cortisol/corticosterone or a synthetic agonist shifts the GR into a conformation with exposed import signals and an increased affinity for co-regulators, thereby enriching the nuclear fraction (Figure 2). Ligand-independent import/export routes also exist, and both the rate and the stability of nuclear accumulation are finely tuned by post-translational modifications (PTMs) of GR (see Section 3).

The functional GR is embedded in a chaperone complex. The key players are classical heat-shock proteins: Hsp90, which is required for activation (its loss markedly compromises the GR-dependent transcription) [123], and Hsp70, which promotes receptor deactivation.

A newly synthesized or ligand-free GR engages Hsp70/Hsp40, which prevents LBD aggregation and maintains a ligand-competent state. The co-chaperone Hop/STIP1 bridges Hsp70 to Hsp90 and loads the GR onto Hsp90, forming the client-loading complex. The ATPase cycle of Hsp90 then takes over [124,125]: ATP hydrolysis closes the Hsp90 “lid,” Hsp70 dissociates, and the co-chaperone p23 stabilizes the closed conformation, which is the client maturation step at which the GR LBD is “dialed in” to a high-affinity ligand-binding state [126]. In the client maturation complex, the GR undergoes conformational changes and restores its activity [127,128]. In practice, more than one Hsp70 contributes: one takes the canonical loading role, and the other acts as a scaffold to remodel the complex. This modular cycle is shared across many Hsp90 clients, with fine-tuning achieved by the specific co-chaperone repertoire [129].

In addition to Hsp90 and p23, the GR chaperone complex directly engages immunophilins, notably FKBP52 [34,35,36] and FKBP51 [37,38,39,40]. These proteins are thought to bind Hsp90 at late stages of client maturation [130] (with FKBP52 replacing FKBP51 upon ligand activation of the receptor) and to exert opposing effects once bound. FKBP51, which has a low affinity for dynein, impedes the nuclear translocation of the complex, whereas the association of the ligand-activated complex with FKBP52 facilitates the nuclear import [131,132,133]. Notably, the expression of the FKBP51 gene is enhanced by GR, providing negative feedback [134,135]. In place of FKBPs, the complex can incorporate CyP40 (cyclosporin A-binding immunophilin) [136] or PP5 (protein phosphatase 5) [137], which, like FKBPs, contain TPR (tetratricopeptide repeat) domains; CyP40 typically augments transcriptional responses, while PP5 can act both as a co-chaperone and as a phosphatase, apparently enabling finer tuning [138,139,140,141,142]. However, the recent literature on these alternatives is relatively sparse, and research attention has shifted strongly toward FKBP51/52. Other proteins can associate with the complex as well, but their roles appear largely auxiliary [40].

Upon ligand binding, the receptor translocates to the nucleus together with its chaperone complex, a process mediated by dynein [143,144] via immunophilin interactions. Transit through the nuclear pore is enabled by importins and nucleoporins. The GR often crosses the pore as part of an Hsp90-containing complex [145,146], which helps preserve the active conformation up to chromatin engagement.

The direct evidence on the GR chaperone complexes in prenatal stress is still fragmentary [147], yet system logic argues for their importance during this period. First, FKBP51 is an early, robust GR target: maternal glucocorticoids can alter its expression in the placenta and fetal tissues, shifting the set point of GR sensitivity from months to years [148,149,150,151]. Second, the expression of the Hsp90 machine components and co-chaperones varies by tissue and developmental stage; the same hormonal pulse can, therefore, produce distinct import kinetics and target gene repertoires. Together with the epigenetic mechanisms discussed below, these features provide a mechanistic substrate for the long-term fetal programming along the stress–metabolism–immunity axis.

## 3. Regulation of GR Activity

The effects of glucocorticoids cannot be reduced to a simple “ligand present-signal on” model. A cell’s sensitivity to a given hormone level is stratified across layers: post-translational modifications of the receptor itself, the composition of co-regulatory complexes, and the oligomeric state and genomic docking sites at which the GR operates. These layers do not act in isolation: modifications reshape co-factor affinity; co-factors remodel chromatin accessibility; and the architecture of the DNA-bound assemblies imposes specific PTM requirements. Consequently, the “same” GR becomes a different regulatory machine across tissues and physiological states.

### 3.1. Post-Translational Modifications: Phosphorylation, Acetylation, SUMOylation, and Ubiquitylation

Phosphorylation: The N-terminal domain of the GR (AF-1) harbors several serine residues whose phosphorylation alters the transcriptional output, the nuclear–cytoplasmic shuttling, and the co-factor interactions. The best that have been studied are Ser203, Ser211, and Ser226 (human GRα numbering). The phosphorylation of Ser211 (including by p38, MAPK, and CDK9) stabilizes the active receptor state and its contact with the co-activators, prolonging the residence time on DNA [151,152,153]. In contrast, Ser226 phosphorylation by JNK kinases enhances the nuclear export and reduces the time window during which the GR can recruit the transcriptional machinery [41,42]; accordingly, the Ser211/Ser226 balance often predicts response strength. Ser203, modulated by CDK2/CDK5, creates biases in the receptor toward activating or repressing complex configurations [152], with the effects contingent on the cellular context and accompanying PTMs. The additional AF-1 serines are engaged by stress signals; the overall logic holds: AF-1 phospho-codes set the receptor’s mode, rather than simply switching it on or off. Table 2 shows the main phosphorylation sites of the GR with a description of the physiological effect. Clinically, the dysregulation of these events contributes to hyper- or hyposensitivity to glucocorticoids in inflammatory diseases, such as asthma [154], and stress-related disorders [155]. For example, under prenatal stress, the systemic inflammation elevates p38 MAPK/JNK activity, increasing Ser226 phosphorylation and accelerating the GR shuttling, which shortens the DNA dwell time and weakens the activation of genes mediating the HPA axis negative feedback.

Acetylation and deacetylation: Upon ligand binding, the GR becomes acetylated within the hinge and AF-1 regions. The classic sites include Lys494/Lys495 (hinge) [159], targeted by the HAT activity of p300/CBP [160] and the circadian CLOCK/BMAL1 [161,162] complex: acetylation lowers the affinity for GREs and facilitates chromatin dissociation [161], effectively narrowing the activation window. Acetylation of Hsp90 further dampens the GR chaperone cycle [163], indirectly reducing ligand-induced activation. Several studies also highlight Lys154 (AF-1), where acetylation correlates with transcriptional competence yet primes the receptor for polyubiquitylation and proteasomal turnover [160], which is a direct example of PTM crosstalk. Deacetylation acts reciprocally: HDACs and SIRT1 prolong the GR DNA residence and enhance the repression of NF-κB [159]. Notably, SIRT1 also augments the GR signaling via protein–protein interactions that are independent of its catalytic activity [164]; thus, it is more accurate to view a tissue- and exposure-dependent shift in the acetyl/deacetyl balance. In perinatal exposure models, HDAC/SIRT1 activity can increase (e.g., with a maternal high-fat diet or stress surrogates), lowering the net acetylation and is associated with an increased risk of affective phenotypes in offspring [165,166,167].

SUMOylation: SUMO adducts retune target gene spectra and operational “modes” of the GR. In the NTD (human Lys277 and Lys293), SUMOylation promotes transrepression, including the suppression of NF-κB/AP-1 via tethering and a direct action at nGREs, while also accelerating receptor turnover and facilitating degradation [168]. Moreover, the anti-inflammatory action of the GR—including both the GC-induced tethered indirect transrepression of NF-κB/AP-1 targets (via corepressor recruitment) and the direct transrepression at negative GREs—depends on the SUMOylation within the NTD [169,170]. In the LBD (human Lys703), SUMO can instead enhance ligand-driven transactivation [171]. Outcomes are site-specific and strongly conditioned by SUMO-interacting motifs (SIMs) within co-regulators (e.g., NCoR/SMRT). Because hypoxia broadly increases cellular SUMOylation [172,173,174], it is plausible, though not yet comprehensively shown, that placental and fetal hypoxia shifts the GR’s SUMO status; the expected direction aligns with a protective “braking” of the pro-inflammatory pathways.

Ubiquitylation and proteasomal control: Ubiquitylation is a principal mechanism that sets cellular GR abundance. The ligand activation is followed by polyubiquitylation and proteasomal degradation, refreshing the GR pool and preventing over-stimulation. The targeting of the GR to the proteasome is mediated by E3 ligases, chiefly CHIP (C-terminus of the Hsc70-interacting protein) [175,176,177], Hdm2/Mdm2 [178], and FBXW7 [179]. During chronic glucocorticoid elevation, E3-ligase activity (notably Mdm2) increases [180], providing additional protection against hyperactivation.

**Figure 3:**
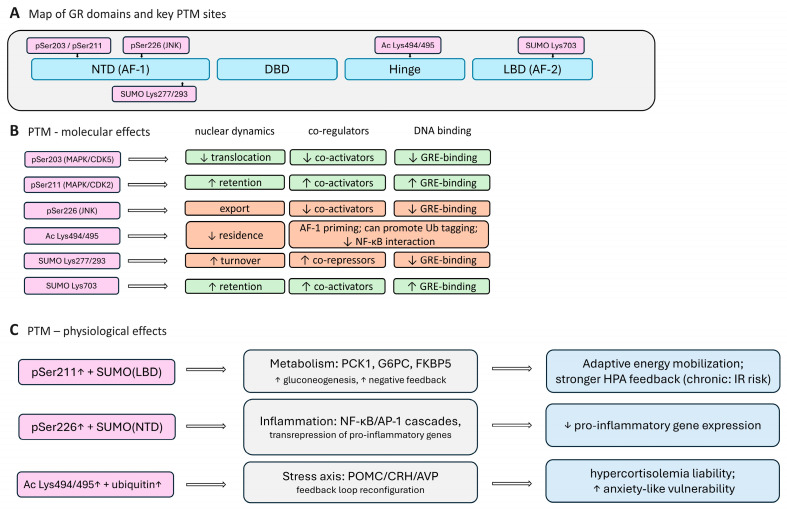
The effects of PTM on GR activity. The post-translational modifications of the glucocorticoid receptor (GR): from domains and PTM sites to molecular actions and physiological outcomes. (**A**) The domain map of the human GR with selected, well-characterized PTM: Ser203/Ser211 in the intrinsically disordered NTD/AF-1; Ser226 (JNK target) within the DBD–hinge junction; Lys494/495 acetylation in the hinge; and SUMO acceptors Lys277/293 (NTD) and Lys703 (LBD/AF-2). (**B**) The site-specific PTMs reprogram the GR behavior at three mechanistic levels. Phosphorylation at Ser203 dampens nuclear import and co-activator engagement; Ser211 phosphorylation stabilizes the active, DNA-bound state; and Ser226 phosphorylation promotes nuclear export and weakens GRE binding. Hinge acetylation (Lys494/495) prolongs chromatin residence, primes AF-1, and can facilitate ubiquitin tagging; SUMOylation at Lys277/293 increases receptor turnover and favors corepressor recruitment/tethering to NF-κB; and SUMOylation at Lys703 in the LBD enhances co-activator docking via AF-2 and supports GRE-driven transactivation. (**C**) The convergent PTM “codes” shape system-level outputs. A Ser211-phospho plus LBD-SUMO state biases the GR toward metabolic gene programs (e.g., PCK1, G6PC, and FKBP51), strengthening negative feedback but chronically increasing the insulin-resistance risk; a Ser226-phospho plus NTD-SUMO state favors the repression of NF-κB/AP-1 inflammatory cascades; and hinge acetylation together with enhanced ubiquitination accelerates receptor turnover and can reconfigure POMC/CRH/AVP feedback, predisposing the offspring to hypercortisolemia and anxiety-like vulnerability.

Thus, post-translational modifications are a central tier in tuning the GR activity, enabling adaptive control of the receptor. The PTMs modulate ligand affinity, stability, subcellular localization, and the capacity to engage co-activators or co-repressors and DNA-binding sites. We presented some of the effects of PTM in Figure 3.

### 3.2. Co-Activators and Co-Repressors: Who Turns Binding into Transcription

The GR regulates gene expression through interactions with specific co-activators and co-repressors that determine the direction of transcriptional activity. Activation requires making chromatin accessible and docking mediator complexes; repression, by contrast, requires chromatin compaction and/or blocking access for other factors. The precise efficiency of the transcription depends on which co-regulators are available in a given cell at a given moment.

***Chromatin-remodeling complexes and histone modifiers***. Initiating transcription from ordered, compact chromatin is difficult; therefore, the GR relies on chromatin-remodeling complexes to render GRE sites more accessible. The most important players that “open” the DNA template for GR are SWI/SNF complex proteins (with BRG1 as its catalytic subunit) [181,182]. These are recruited by the ligand-activated GR [183] and subsequently enable its transcriptional activity [184]. Conversely, BRG1 can recruit the GR together with histone deacetylases (HDAC2) to promoters that must be trans-repressed [185] rather than activated (e.g., *pomc*) or otherwise participate in gene repression [186].

Chromatin modifiers (histone acetyl- and methyl-transferases, deacetylases, and demethylases) play analogous roles to remodelers. Histone acetyltransferases (HATs) add acetyl groups to histones (chiefly H3 and H4), reducing their positive charge and weakening their electrostatic interactions with DNA. The resulting chromatin loosening, akin to SWI/SNF action, facilitates access for nuclear receptors and their co-regulators. The principal HATs associated with the GR are p300/CBP [187,188,189]; ACTR [190] and the recruited p/CAF [191] are acting synergistically, which also has HAT activity.

Histone methyltransferases (HMTs) have a broader spectrum of possible outcomes, as histone methylation can be either activating or repressive. For example, Suv4-20h1, when bound in a complex containing GRIP1 [192], suppresses GR-induced transcription. In contrast, the methyltransferases CARM1 and PRMT1 act as co-activators when assembled with the same GRIP1 [193,194]. The synergistic effects can also come from another methyltransferase, G9a (a lysine-specific enzyme, which is in contrast to the arginine-specific enzymes above) [195,196].

Histone deacetylases (HDACs) are most often recruited by the GR into repressive complexes; their activity deacetylates not only histones [197]—condensing chromatin and reducing the accessibility to other transcription factors—but also the GR itself [159]. Nevertheless, HDAC1 can function as a co-activator, but only in an acetylated state in which its deacetylase activity is inactivated [198]—serving then as a scaffold rather than a chromatin modifier. HDAC2 is a classical co-repressor of pro-inflammatory genes (helping the GR to repress NF-κB activity) [159]. HDAC3 works synergistically with NCoR/SMRT and likewise acts most often as a co-repressor [199,200,201].

Among demethylases, one of the best that has been characterized is LSD1 (KDM1A). LSD1 is recruited to enhance the GR-dependent genes and selectively remove H3K4me2 marks, which is necessary for the GR-mediated activation [202]. At the same time, the GR can control the transcription and stability of demethylases themselves [203,204], greatly complicating their interplay.

***Nuclear receptor co-activators (NCOAs)***. The key co-activators include members of the p160 family: SRC-1 (NCOA1) [205] and TIF2/GRIP1 (NCOA2) [206]. They contain characteristic LXXLL motifs and possess acetyltransferase activity, supporting chromatin loosening and transcriptional activation. Another family member includes SRC-3 (ACTR/CIP/AIB1/RAC3) [207], which can reduce the GR’s anti-inflammatory effect by competing with it for binding to NF-κB. Beyond its intrinsic HAT activity, NCOA1 recruits additional HATs (p300/CBP, p/CAF) [187,208], further acetylating histones and decreasing chromatin density. CCAR1 serves as the bridge between the GR, the p160 co-activators, and direct transcriptional activation and is required to recruit a mediator (the DRIP/TRAP complex) and RNA polymerase II [209]. NCOA2 participates not only in the GR-dependent activation but also in tethered repression (e.g., the tethering of NF-κB and AP-1) [210,211] and does so at multiple stages of transcription [212], making GRIP1 a key mediator of glucocorticoids’ anti-inflammatory function. Another function of TIF2/GRIP1 is the recruitment of methyltransferases CARM1 and PRMT1 [193,194] as secondary co-activators, as well as Suv4-20h1 [192], which diminishes the GR transcriptional activity. Because SRC-1 and SRC-2 exert similar effects on the GR signaling, the reduced expression of one can be partly compensated by the increased expression of the other [213].

***Nuclear receptor co-repressors (NCoRs) and SMRT***. Unlike NCOAs, these proteins participate primarily in GR-dependent repression [214]. They are required for IR nGRE–mediated repression, acting via the assembly of repressive complexes and the recruitment of HDACs (particularly HDAC3) [169,199], although evidence for their necessity in the repression of pro-inflammatory genes has been somewhat contradictory [215].

Co-regulators play a critical role in modulating the GR signaling: depending on their composition and abundance, the same receptor, activated by the same ligand, can cause opposite effects on the transcription of the same gene. For example, the *crh* gene in the PVN of the hypothalamus is negatively regulated by the GR, which is an integral part of the HPA axis feedback loop. However, in the limbic system, *crh* expression increases under the GR control, which is attributable to differences in co-regulators: SRC-1a (acting here as a co-repressor) predominates in the PVN, whereas SRC-1e (a co-activator) predominates in the amygdala and hippocampus [216,217,218]. Several other genes are also regulated by the GR in a tissue- or context-specific manner, though the differences are less dramatic. Thus, transcription of *pomc* in the pituitary is clearly repressed by the GR [2], yet adrenalectomy decreases *pomc* expression in the hypothalamus [219,220], i.e., the GR activation may not directly stimulate transcription in that context but exerts a permissive function. Similarly, elevated glucocorticoid levels can lead, depending on the tissue and the duration of exposure, to either an increased or decreased GR-dependent expression of BDNF [1,221,222,223].

### 3.3. Dimerization and DNA Engagement: From GREs to Composite Sites

The most canonical action of the GR is binding to GREs or nGREs as a homodimer, although the sequence of oligomerization versus DNA engagement remains under discussion, with evidence supporting both possible orders of events [224,225,226]. At the same time, the monomeric GR is thought to participate predominantly in transrepression by interacting with other transcription [227,228] factors such as NF-κB or AP-1 (though the necessity of tethering is now [229,230,231] being questioned in favor of direct DNA binding at “cryptic” sites), thereby suppressing the expression of pro-inflammatory genes, or by directly binding IR-nGREs [232] (inverted-repeat nGREs). In addition, monomers can engage standalone half-GREs [233] as well as half-GREs that are embedded within composite promoters (promoters in which a GRE lies adjacent to the binding site of another transcription factor) [234,235]. Notably, some reports [211] suggest that the GR monomers lack physiological relevance (a view that upends the classical model), whereas higher-order structures—tetramers and oligomers [236]—may be more consequential than previously appreciated.

The GR can also form heterodimers. For example, the formation of an AR/GR dimer could, in principle, lead to the mutual inhibition of both receptors’ activities [237]; by contrast, when dimerization-deficient mutants are co-expressed, such inhibition does not occur. Perhaps best studied are GR/MR dimers [238] because, as noted earlier, both receptors can be activated by glucocorticoids, and the GR/MR expression pattern differs from that of either homodimer [239,240]. Acute stress and pulsatile glucocorticoid exposure have been shown to enhance heterodimer formation [241,242], potentially broadening the GR’s capacity to regulate the stress response, although the physiological role of heterodimerization remains incompletely defined.

## 4. Epigenetic Regulation of GR

**Figure 4:**
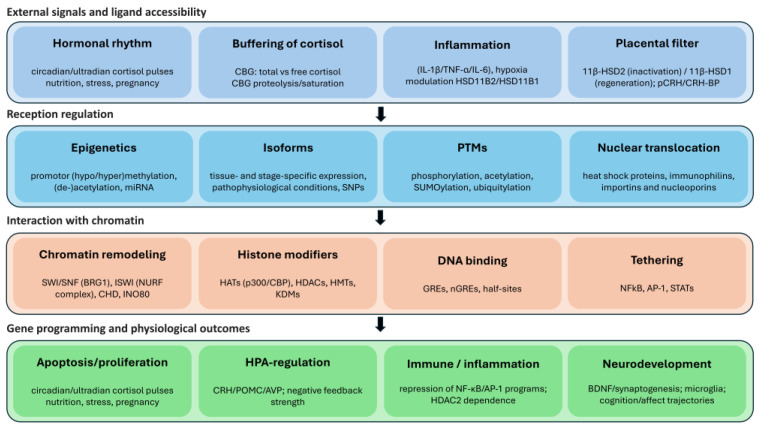
Multilevel regulation of the GR function. Top tier (external signals/ligand accessibility): circadian–ultradian cortisol pulses, nutritional/stress inputs, and pregnancy alter free cortisol via CBG buffering; inflammatory cytokines modulate signaling; the placental filter (11β-HSD2 inactivation vs. 11β-HSD1 regeneration; pCRH/CRH-BP) sets fetal exposure. Second tier (reception regulation): tissue- and stage-specific responsiveness is set by epigenetics (promoter/enhancer methylation, (de)acetylation, miRNA), receptor isoforms, post-translational modifications (PTMs), and chaperone-guided nuclear translocation (Hsp70/90, immunophilins, importins/nucleoporins). Third tier (interaction with chromatin): the GR accesses DNA through remodelers (SWI/SNF/BRG1) and histone modifiers (HATs p300/CBP, HDACs), binds GREs/nGREs/half-sites, or represses via tethering to NF-κB, AP-1, or STATs. Bottom tier (gene programming/physiological outputs): context-specific programs drive apoptosis/proliferation, the HPA axis regulation (CRH/POMC/AVP; feedback strength), repression of inflammatory genes (HDAC2-dependent), and neurodevelopmental trajectories (BDNF/synaptogenesis; microglia), providing a framework for how prenatal stress perturbs nodes across the cascade.

The glucocorticoid receptor is not merely a protein, but the output hub of a larger regulatory system in which chromatin sets the accessibility, DNA and histone marks encode memory, and noncoding RNAs provide the fine-tuning. For *nr3c1*, this multilayered control (Figure 4) is especially salient: the very same hormonal pulse elicits distinct transcriptional programs in the liver, hippocampus, and placenta precisely because each compartment inhabits its own epigenetic landscape [3]. The epigenetic regulation of *nr3c1* defines the language by which different tissues “read” an identical hormonal signal. Under physiological conditions, this language is flexible: the choice among alternative first exons and promoters of *nr3c1* sets 5′UTR architecture and baseline transcription; the chromatin state around glucocorticoid response elements (GREs), from histone marks to nucleosome positioning, governs regulatory accessibility; non-coding RNAs and RNA-binding proteins sculpt transcript fate; and post-translational modifications (PTMs) of the GR bias the balance between transactivation and transrepression. The daily cortisol oscillations synchronize these layers: circadian complexes such as CLOCK/BMAL1 modify histones near GREs and acetylate the GR hinge [161,162], so the same hormonal input can elicit distinct transcriptional outputs across the day. Under prenatal stress, which encompasses inflammation, hypoxia, and hormonal fluctuations, this finely tuned system is displaced and then stabilized as epigenetic memory, retuning the HPA axis negative feedback and immune tone in the offspring [3,4].

The best studied mechanism is DNA methylation within the 5′ regulatory region of *nr3c1*. Because transcription of the human gene is controlled by a set of alternative first exons with their own promoters, it is more accurate to consider which promoter variant and enhancer neighborhood are active in a given tissue and developmental period than to refer to “*nr3c1* promoter methylation” in the abstract. In humans, *nr3c1* is governed by a set of alternative first exons (“exon 1” variants) with their own promoters [243,244]. These segments do not alter the GR amino-acid sequence, but they determine which 5′UTRs are variant and which promoters are active in a given tissue and developmental period [244]. As a rule, higher 5mC density across these regulatory modules corresponds to reduced *nr3c1* transcriptional activity, but the magnitude of the effect depends on which promoter variant is active, whether nearby sites for “companion” factors (C/EBP, AP-1, etc.) are present, and how promoter–enhancer looping is organized. DNMT1 maintains established 5mC patterns, while DNMT3A/B lay down new ones [245,246]. Prenatal exposure to inflammation, hypoxia, and hormonal fluctuations can shift DNMT activity in the placenta and in the developing brain, increasing methylation at particular *nr3c1* promoter variants and their enhancers [247,248,249,250]. Phenotypically, this often presents as a reduced GR expression, a weakened HPA axis negative feedback, and a greater variability in stress responsiveness in offspring [5,6,7,8,9]. The chromatin context determines not only *nr3c1* transcript levels but also how an activated GR can execute its programs [251]. Loci primed for activity typically carry H3K4me1/H3K27ac, whereas silent domains bear H3K27me3 or H3K9me2/3; accordingly, the GR binding rarely begins de novo [251,252]. The SWI/SNF remodeler with BRG1 physically repositions nucleosomes to enhance the access to GREs and composite elements [253], while p300/CBP and p/CAF increase H3/H4 acetylation and further loosen chromatin [188,190]. In other settings, the same machinery assembles into repressive complexes with HDAC2/HDAC3 and NCoR/SMRT, sustaining suppression of pro-inflammatory genes and implementing IR-nGRE-mediated repression [159,201]. At tissue-specific enhancers, the GR frequently cooperates with assisted pioneer factors such as C/EBPβ or FOXA1, explaining why identical GRE motifs are active in the liver yet remain silent in neurons [254,255].

MicroRNAs provide a rapid, context-sensitive layer of control over glucocorticoid signaling. In neurons and the placenta, miR-124 and miR-18a recognize conserved sites within the *nr3c1* 3′UTR, reducing mRNA stability and translation, thereby lowering the GR abundance [43,44]. Beyond direct *nr3c1* targeting, miRNAs reconfigure the GR output by acting on co-regulators (e.g., *fkbp5*) and steroid-metabolizing enzymes (e.g., *hsd11b1*), biasing the system toward canonical transactivation or SUMO-dependent transrepression without altering the receptor’s coding sequence [256,257,258]. The magnitude and direction of these effects are strongly context-dependent: alternative polyadenylation remodels 3′UTR length and the density of miRNA binding sites [259,260], while RNA-binding proteins (e.g., HuR) can mask or expose these sites, imparting cell type and stage-specific responses [261]. The placenta adds an inter-tissue conduit: trophoblast miRNAs, particularly from the C19MC cluster, are packaged into exosomes and released into maternal and fetal circulations, where they can influence *nr3c1* expression and neuro-immune programs in sensitive windows; several placental miRNA profiles exhibit sex-biased expression, suggesting a mechanism for sexually dimorphic fetal programming [262,263,264].

The PTMs of the GR set their operating mode and co-regulator selectivity. Serine phosphorylation within AF-1 confers distinct kinetic and functional properties: Ser211 phosphorylation is associated with nuclear retention and a prolonged DNA residence with robust transcriptional activity, whereas Ser226 phosphorylation by JNK kinases accelerates the nuclear export and shortens the chromatin-bound window [41,42,152,153]. Acetylation of the hinge region by p300/CBP and the CLOCK/BMAL1 complex facilitates GR dissociation from the DNA; deacetylases, including SIRT1 and HDACs, act oppositely and extend the receptor dwell time [159,160,161,162,163]. SUMOylation of N-terminal lysines strengthens transrepressive programs, including the suppression of inflammatory cascades via the NCoR/SMRT–HDAC3 axis and tethered interactions with NF-κB/AP-1, whereas SUMO modifications within the LBD can, in certain contexts, support transactivation through the AF-2 interface [160,164,165,166,167]. Collectively, these PTM codes remodel the affinities for co-regulators and nuclear transport machinery, alter DNA residence times, and redistribute target-gene repertoires, yielding phenotypes that range from altered stress responsivity to shifts in the steroid sensitivity of immune cells.

Deacetylase-mediated regulation constitutes an additional control circuit, particularly in immunity. HDAC2 and HDAC3 are essential for full GR-dependent repression, including anti-inflammatory programs; under oxidative stress, typical of perinatal inflammation, reduced HDAC2 activity impairs the shutdown of inflammatory genes and contributes to the relative steroid resistance [197,198,199,200,201]. Other family members, such as HDAC4 and class IIa/III deacetylases [265,266], help encode tissue differences in glucocorticoid responses and, under prenatal stress, can shift *nr3c1* expression and the profiles of its downstream targets [267,268].

Together, the promoter-specific methylation of *nr3c1*, the reprogramming of partner enhancers (e.g., FKBP5), the shifts in GR PTM codes, and the miRNA circuits create a durable retuning across systems. In the nervous system, the HPA negative feedback is weakened and affective–cognitive trajectories shift; in the immune system, loss of HDAC2 activity and higher GRβ proportions reduce the anti-inflammatory efficacy of steroids; in metabolic and vascular organs, the tissue-level amplification of glucocorticoids via 11β-HSD1 and sympathetic renin priming promotes insulin resistance and elevated blood pressure. Heterogeneity across cohorts is expected because outcomes depend on tissue, sex, gestational window, circadian phase, and genetic modifiers. The epigenetic mechanisms do not switch a gene on or off once and for all. Rather, they open and partially close specific regulatory windows, thereby setting the range within which the GR can operate in response to pulsatile cortisol fluctuations. The diurnal rhythm of the hormone also synchronizes the modifying machinery: circadian complexes (e.g., CLOCK/BMAL1) regulate the acetylation of both histones and the GR itself, and DNA methyltransferases (DNMTs) display rhythms and context-dependent shifts, although in humans these are less unequivocal than in model organisms.

***How prenatal stress translates these mechanisms into long-term effects***. Perinatal exposures rarely act through a single lever. Maternal stress elevates cortisol and inflammatory mediators, alters placental blood flow [17,18], and shifts activity across DNMT/TET, HAT/HDAC, and SUMO cascades [169,170]. In the placenta, this is accompanied by a remodeling of marks at the regulatory regions of *nr3c1* and steroid-metabolizing enzymes (e.g., 11β-HSD2), thereby changing hormone availability to fetal tissues [269,270]. In the brain and immune system of the offspring, patterns of 5mC/5hmC at *nr3c1* promoters/enhancers and partner genes are reconfigured; in parallel, ncRNA profiles push the transcript toward a reduced stability [7,271]. At the phenotypic end, this yields attenuated HPA axis feedback, heightened stress reactivity, and shifts in immune balance (Th1/Th2, Treg) [272] that, in adulthood, manifest as an increased risk for being on the anxiety–depression spectrum and for inflammatory conditions [273]. Importantly, the direction of effect depends on the window of vulnerability, tissue, fetal sex, and genetic background.

The translational message follows the same mechanistic logic. Reducing maternal stress, inflammation, and hypoxia; optimizing sleep and circadian alignment; and carefully timing antenatal glucocorticoids act directly on the placental pCRH loop and the 11β-HSD2/11β-HSD1 balance [19,274,275]. The experimental avenues—which targeted the epigenetic editing [276] of *nr3c1* promoter modules and FKBP5 enhancers [277,278], and the modulation of placental miRNAs [279] and their delivery—are promising but demand strict developmental safety and a careful alignment with windows of vulnerability. This perspective links transient prenatal exposures to durable phenotypes through a coherent causal chain and, at the same time, delineates realistic points for prevention and therapy.

The epigenetic regulation of *nr3c1* is a field of interactions, not a single mechanism. The DNA marks on alternative promoters, the histone code and remodeling machinery, the three-dimensional promoter–enhancer contacts, and the ncRNA layer together determine how much and how quickly a cell “opens the door” to the GR signaling. Prenatal exposures shift these settings so that the same hormonal pulse is interpreted differently months and years later. The epigenetic changes to *nr3c1* that are induced by stress in the prenatal and early postnatal periods can have long-term consequences for neural and immune function. A low GR expression due to epigenetic modification fosters heightened stress sensitivity, cognitive impairment, and the development of anxiety–depressive disorders in offspring. In addition, an altered GR regulation can skew immune responses, raising the risk of autoimmune and inflammatory diseases in adult life.

Thus, the epigenetic regulation of *nr3c1* is a crucial mechanism of adaptation to stress; when perturbed by adverse early-life environments, it can contribute to the development of pathology.

## 5. Transplacental Passage of Maternal Glucocorticoids and Effects on the Fetus

### 5.1. Placental Barriers: 11β-HSD2, 11β-HSD1, and Transcortin (CBG)

The placenta is not a mere mechanical filter; it is an endocrine–enzymatic hub that doses and formats maternal hormonal signals for the fetus. The fetus is protected from maternal glucocorticoids primarily by 11β-HSD enzymes [91], which are expressed at the maternal–fetal interface of the placenta, most notably in syncytiotrophoblasts [280]. In humans and rats, there are two 11β-HSD isoforms. 11β-HSD1 converts cortisone to active cortisol, whereas 11β-HSD2 catalyzes the rapid inactivation of glucocorticoids (cortisol and corticosterone) to inert 11-keto forms (cortisone and 11-dehydrocorticosterone) [91]. Therefore, 11β-HSD2 is the principal “filter” on the maternal–fetal glucocorticoid axis. Under stress, inflammation, or hypoxia, 11β-HSD2 expression can decline, allowing more active cortisol to traverse the placenta and act on the fetus [281,282,283]. This is particularly hazardous during critical windows of brain development.

Reductions in placental 11β-HSD2 under stress, inflammation, or hypoxia arise from interlinked molecular and epigenetic mechanisms. A well-characterized driver is the action of pro-inflammatory cytokines such as IL-1β, TNF-α, and IL-6, which activate transcriptional cascades (e.g., NF-κB) that repress HSD11B2 transcription [269]. Inflammation can also exacerbate oxidative stress, further diminishing enzyme activity [284,285,286,287]. Hypoxic conditions in the placenta reduce the expression of key transcription factors such as SP1 [288] and EGR1 that are normally required to activate the HSD11B2 promoter, thereby lowering 11β-HSD2 expression and increasing fetal exposure to active cortisol. With chronic stress or hypoxia, HSD11B2 promoters may become hypermethylated, rendering them less accessible; this frequently involves the recruitment of DNMT1 and HDACs, which compact chromatin and suppress transcription [289,290]. For example, in preeclampsia and intrauterine growth restriction (IUGR), substantial reductions in 11β-HSD2 activity, driven by placental hypoxia and inflammation, elevate fetal glucocorticoid levels and perturb development [291,292].

By contrast, 11β-HSD1 exerts the opposite effect by regenerating cortisol from cortisone [91]. Under normal conditions, its placental activity is low, but it can rise in certain pathologies, amplifying the glucocorticoid action. In inflammatory states, for instance, in preeclampsia or intrauterine infection, signaling cascades (NF-κB, JAK/STAT) can enhance HSD11B1 transcription [293,294,295]. This is supported by studies showing increased 11β-HSD1 expression in the labyrinth zone of the placenta during inflammation and immune-cell activation [296]. Maternal obesity and gestational diabetes are likewise associated with heightened 11β-HSD1 activity [297], raising local concentrations of active glucocorticoids in the placenta and, accordingly, their passage to the fetus, which is an effect linked to the chronic inflammation and metabolic dysregulation characteristics of these conditions. Paradoxically, glucocorticoids themselves can upregulate 11β-HSD1, particularly under chronic exposure, which is an autocatalytic loop that sustains tissue sensitivity by local cortisol regeneration [298]. During pregnancy, this may be especially problematic, further intensifying fetal exposure. In models of stress and metabolic disturbance, hypomethylation of the HSD11B1 promoter accompanies its overexpression in the placenta.

Beyond enzymes, an upstream systemic buffer operates at the entrance to the placenta: corticosteroid-binding globulin. Most maternal cortisol circulates in a bound state; only the free fraction diffuses into the syncytium. During pregnancy, estrogens increase *serpina6* expression in the liver, boosting CBG synthesis and buffering cortisol fluctuations [15,16]. It is appropriate to view this regulation as a systemic “pre-filter” that reduces the amplitude of hormones that have access to the placenta, while local trophoblast enzymes set the final spatial and temporal selectivity. (Transporters such as P-gp/ABCB1 contribute primarily to certain synthetic glucocorticoids [299], not to cortisol.) A practical corollary is that betamethasone and dexamethasone are poor substrates for 11β-HSD2 and thus cross to the fetus relatively freely during antenatal therapy [300,301]. This underlies their clinical efficacy in promoting lung maturation and, at the same time, necessitates careful dosing and timing of administration.

### 5.2. Regulation of Glucocorticoid Levels in Mother and Fetus

**Figure 5:**
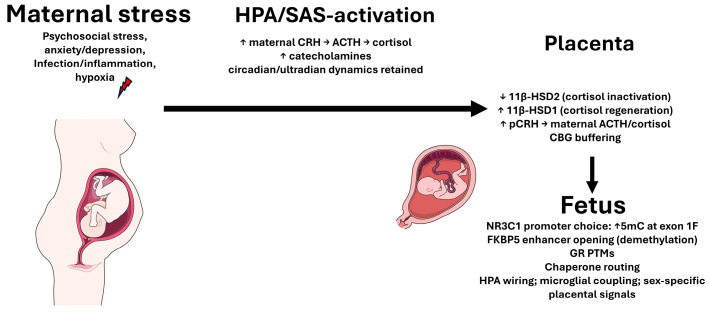
The regulation of the glucocorticoid level in the mother and fetus. The schematic of the maternal–placental–fetal axis controlling fetal exposure to glucocorticoids. On the maternal side, the HPA axis (CRH → ACTH → cortisol) is modulated by circadian/ultradian pulses, stress, nutrition, and pregnancy-related rises in estrogens that increase hepatic CBG (SERPINA6), buffering free cortisol. The placenta-specific signals further shape the input: placental CRH (pCRH) escalates in late gestation and, unlike hypothalamic CRH, is stimulated by cortisol, creating a positive-feedback loop (cortisol → pCRH → ACTH/cortisol). At the maternal–fetal interface, syncytiotrophoblast enzymes determine local hormone availability: 11β-HSD2 rapidly inactivates cortisol/corticosterone to 11-keto forms, whereas 11β-HSD1 can regenerate active cortisol; ABC transporters (e.g., ABCB1/P-gp) limit the transfer of some synthetic glucocorticoids. Therefore, fetal exposure reflects the balance between maternal production/buffering and placental metabolism, which varies with gestational age, inflammatory/hypoxic stressors, and fetal sex. On the fetal side, a gradually maturing HPA axis and tissue-specific GR/MR programs translate the glucocorticoid signal into developmental effects, with critical windows of heightened vulnerability. The figure was created using Servier Medical Art (https://smart.servier.com/), licensed under CC BY 4.0 (https://creativecommons.org/licenses/by/4.0/ (accessed on 10 August 2025)).

The input amplitude of maternal glucocorticoids varies with the circadian rhythm, stress, nutrition, and gestational time [302]. As estrogens and CBG rise, total maternal cortisol increases, but the free fraction does not change linearly [303]. The excess glucocorticoid delivery to the fetus during critical developmental periods induces long-term epigenetic changes [304,305,306]. These periods in embryogenesis are defined windows during which cell differentiation and organogenesis proceed with maximal intensity and are decisive for proper structure and function. During these windows, fetal growth and development are especially susceptible to environmental influences. In early pregnancy, the fetal brain is extraordinarily plastic yet vulnerable to environmental fluctuations that can shape long-term programming [307,308,309]. By the end of week 8 of embryonic development, structures such as the hippocampus, neocortex, and hypothalamus take form, and major divisions of the central and peripheral nervous systems are specified [310]. The first trimester is particularly sensitive: exposures such as stress, inflammation, and undernutrition can drive epigenetic changes associated with the altered development of the limbic system, manifesting later as heightened anxiety, reduced stress adaptability, and increased risks of depression and ADHD [300,301,302,303,304,305,306,307,308]. In such a milieu, the enzymatic barriers must operate with a particular precision. The placental CRH in the third trimester further stimulates the maternal HPA axis, establishing a positive feedback loop, cortisol → placental CRH → ACTH/cortisol [311]. In Figure 5, we present a generalized diagram of the transit of maternal glucocorticoids into the fetus.

On the fetal side, the fetal HPA axis progressively “switches on”: the adrenal glands ramp up steroidogenesis, and the brain begins to establish feedback loops. Under normal circumstances, local inactivation within the placenta ensures that the maternal external signal does not drown out the fetus’s nascent regulatory circuitry. When 11β-HSD2 weakens, and/or 11β-HSD1 strengthens, the fetus receives a louder and more prolonged glucocorticoid pulse, mismatched to its maturational stage. Against this backdrop, the synthetic glucocorticoids (at therapeutic doses) act as strong forcing factors, effectively bypassing the placental protection.

Sex, gestational age, and comorbid states (e.g., obesity, infection) shift the equilibrium. Some groups have reported sex differences in 11β-HSD2 expression and in sensitivity to inflammatory cues; closer to the time of birth, portions of the placental barrier physiologically relax. These nuances are crucial for interpreting both experimental and clinical data.

### 5.3. Epigenetic Impact of Maternal Hormones on the Fetus: Windows of Vulnerability and Targets

When active glucocorticoids traverse the placenta, they act not only as acute signals but also as programmers of future responses. The substrate is their interaction with the epigenome of the developing brain and immune system. In early ontogeny, from the first trimester onward, there is an intense construction of the limbic circuits, the hypothalamus, and the cortex, the formation of neuron–glia networks, the maturation of microglia, and the emergence of immune competence. During these windows, excessive glucocorticoid exposure increases the likelihood of stable shifts at regulatory regions of genes that govern the stress-response axis.

Among the vulnerable targets is *nr3c1*. The elevated methylation of specific promoter variants (e.g., segments corresponding to “exon 1F” in human cohorts) is associated with reduced GR expression and weakened HPA-axis negative feedback in newborns whose mothers experienced severe stress during pregnancy. In parallel, the epigenetics of *nr3c2* and partner genes (e.g., FKBP5) is altered, as is the profile of placental miRNAs, including neuronally relevant miR-124/miR-18a, which is capable of reducing the *nr3c1* transcript stability/translation in neurons and immune cells. Some of these RNAs are delivered via exosomes, adding an intertissue “courier” layer to the hormonal signal [7,269,270,271,272,273].

Glucocorticoids also reshape the histone landscape: by recruiting HAT/HDAC complexes and methyltransferases, they rewrite acetyl and methyl marks as the enhancers and promoters of the genes controlling neuroplasticity (including BDNF), synaptogenesis, and immune tolerance. In an inflammatory milieu, SUMO-dependent transrepression by the GR is strengthened, shifting the balance from activation toward the suppression of target genes and cementing an anti-inflammatory, yet potentially over-braked, tone during critical developmental periods [159,160,161,162,163,167,168].

Phenotypically, such reprogramming manifests as heightened stress reactivity, altered cognitive trajectories and affective vulnerability (anxiety–depressive features and ADHD-like traits), and shifts in immune balance (Th1/Th2, Treg), thereby increasing the risk of inflammatory and autoimmune conditions later in life [272].

The placental control of glucocorticoids reflects the coordinated action of the systemic buffer (CBG), the local enzymatic barrier (11β-HSD2/11β-HSD1), and the trophoblast’s epigenetic program. When the environment shifts this balance—via inflammation, hypoxia, metabolic stress, or pharmacologic intervention—the fetus receives a different hormonal experience that is memorized by the epigenome and alters the behavior of the stress–immunity–metabolism axis for years to come. In the next chapter, this logic will be expanded to the entire fetal HPA axis: how prenatal stress rewrites regulation of CRH/ACTH/cortisol and redistributes the roles of the GR/MR across tissues.

## 6. Prenatal Stress and Glucocorticoid System Disorders

Prenatal stress is not one factor but a constellation of psychoemotional, hypoxic, and infectious influences that reconfigure the maternal–placental–fetal axis so that the fetal glucocorticoid signal becomes louder and more prolonged than the fetus’s maturational stage permits. The central logic is straightforward: maternal stress activates the mother’s HPA axis [312] and sympathoadrenal system [313,314]; the placenta, with its own endocrine circuitry, amplifies portions of these signals; the placental enzymatic barriers (chiefly 11β-HSD2) weaken; and the developing brain and immune system of the fetus are then subjected to an excessive hormonal and inflammatory load, which is epigenetically inscribed.

### 6.1. CRH, ACTH, Cortisol: How Hyperactivation Is Initiated and Sustained

Stress signals in the mother increase CRH secretion by the hypothalamus, ACTH rises in the anterior pituitary, and the adrenal glands ramp up cortisol production. Against this backdrop, the placenta adds its own contribution: placental CRH expression increases via positive feedback from cortisol (in contrast to hypothalamic CRH, which cortisol suppresses), establishing a CRH–cortisol loop that sustains hypercortisolemia. In parallel, the sympathoadrenal system is activated: norepinephrine and epinephrine cause the vasoconstriction of uteroplacental vessels via α_1_-adrenoceptors, reducing perfusion and aggravating hypoxia, and β_2_-mediated vasodilation during the acute stress phase generally does not compensate for the spasm [314].

### 6.2. Dysregulation of the GR in the Offspring: From Exposure to Epigenetic Memory

Excess glucocorticoid signaling during sensitive periods not only shifts short-term transcription but also rewrites the set points of the stress-response axis. At the receptor level, this manifests as a rebalanced GR/MR ratio and isoform distribution, altered repertoires of co-regulators and enzymes of pre- and post-translational modification, and an epigenetic retuning of genes within feedback circuits.

The convergent evidence from independent laboratories supports the idea that prenatal adversity can permanently reshape the GR/MR signaling states via the epigenetic regulation of receptor programs. In gestational hypoxia models, reduced GR abundance in the developing brain has been linked to increased DNA methylation and impaired binding of transcription factors at the GR promoter regions, with functional consequences such as heightened vulnerability to hypoxic–ischemic injury [315]. Similar hypoxia-driven promoter hypermethylation and repression of GR transcription has been demonstrated in the fetal heart, where it programs increased ischemia sensitivity in postnatal life [316]. In prenatal stress paradigms, the sex-dependent MR and GR expression patterns and the fetal MR/GR ratio that were observed under control conditions can be attenuated or lost, which is consistent with receptor-level retuning during sensitive windows [317,318].

The experimental models illustrate causality. For example, in our prior rat studies, hypoxic stress on gestational days 14–16 produced persistent changes in chromatin epigenetic marks in the offspring’s brain [319], reduced glucocorticoid sensitivity of extra-hypothalamic structures due to the downregulated GR expression, and diminished efficiency of the GR-dependent transcription [320]. This effect is mediated by the maternal glucocorticoid response to hypoxia and subsequently leads to impaired glucocorticoid negative feedback, hyperproduction of corticosterone, and both visceral disturbances of glucocorticoid-dependent processes and neurological abnormalities, including a depression-like phenotype, vulnerability to addictive behavior, dysfunction of the glutamatergic system, endocrine and metabolic derangements, cognitive deficits, premature neuronal loss, and a state resembling accelerated aging [22,320,321,322]. The related prenatal stress models further indicate that long-term glucocorticoid phenotypes can emerge through both GR-dependent and GR-adjacent routes. For example, prenatal stress in mice has been shown to induce persistent alterations in dentate gyrus development, hippocampal neurogenesis, and HPA axis regulation, which is consistent with glucocorticoid-linked developmental reprogramming even when the hippocampal GR abundance is not the only altered node in the circuit [304,323,324]. In parallel, the placental “barrier” mechanisms and stress-related co-regulators, including HSD11B2, NR3C1, FKBP5, and transport-associated pathways, exhibit stress-associated epigenetic changes that have been linked to altered offspring glucocorticoid regulation and an elevated corticosterone, supporting a multi-compartment model in which the placenta and the brain jointly contribute to long-term GC phenotypes [147,325,326,327,328]. In a chronic unpredictable prenatal stress model in mice, offspring showed impaired spatial memory, reduced histone acetylation (AcH3Lys14), increased DNMT1, and elevated basal corticosterone, which were consistent with the long-term reprogramming of the HPA axis [23]. The independent studies using chronic unpredictable prenatal stress paradigms similarly report durable cognitive and affective phenotypes accompanied by epigenetic signatures that are consistent with reduced transcriptional accessibility in the hippocampus. In mice, chronic unpredictable prenatal stress has been associated with impaired spatial memory and sex-sensitive increases in DNMT1 alongside reductions in histone H3 acetylation, together with elevations in circulating corticosterone—especially in females—supporting a coupling between the baseline GC tone and the epigenetic repression [23]. In rats, maternal chronic stress during pregnancy has likewise been associated with offspring learning/memory deficits, which were accompanied by reduced hippocampal BDNF/Arc expression and elevated corticosterone [329]. Behaviorally, prenatal adversity has also been linked to altered reward-related phenotypes, providing an external line of evidence consistent with later vulnerability to addictive behavior. For example, prenatal stress exposure has been reported to increase an adult’s vulnerability to the reward of cocaine in conditioning paradigms, even when pubertal anxiety-like measures are largely preserved [330]. Mechanistically, gestational immune challenges can modify the expression of GR regulators such as FKBP5 across multiple stress-sensitive brain regions, indicating that co-chaperone-mediated changes in GR responsivity may be generalized beyond purely “psychological” stress models [331]. Finally, human placental data support an analogous pathway: trimester-specific maternal anxiety can be associated with sex-dependent placental FKBP51 expression, and FKBP51 can mediate the associations with birthweight and neonatal cortisol measures, linking prenatal distress to early growth/endocrine outcomes via placental glucocorticoid signaling modulators [147].

The chronic hyperactivation of the HPA and sympathoadrenal systems also suppresses the hypothalamic–pituitary–gonadal (HPG) axis [20,21]. The cortisol and catecholamines inhibit hypothalamic GnRH secretion, reducing pituitary LH and FSH output. This disrupts gonadal steroidogenesis: in males, Leydig cell numbers decline, and in females, the estrous cycle is perturbed [24]. The sex differences are not incidental but systemic modifiers [25]. In male rats, prenatal hypoxia reduces placental HSD11B2 more strongly, and in adulthood, they show a higher-amplitude corticosterone response to stress. These differences align with placental sexual dimorphism and the disparities in maturation rates of regulatory axes. Finally, some effects extend beyond one generation. In certain studies, men exposed to prenatal stress exhibited hypomethylation of the *nr3c1* promoter in their sperm, which is consistent with the notion of a transgenerational transmission of vulnerability [26,27]. Mechanistically, changes in microRNAs and the epigenetic marks in oocytes may contribute as well, though the body of robust evidence is currently smaller than what exists for the sperm and warrants cautious interpretation.

**Figure 6:**
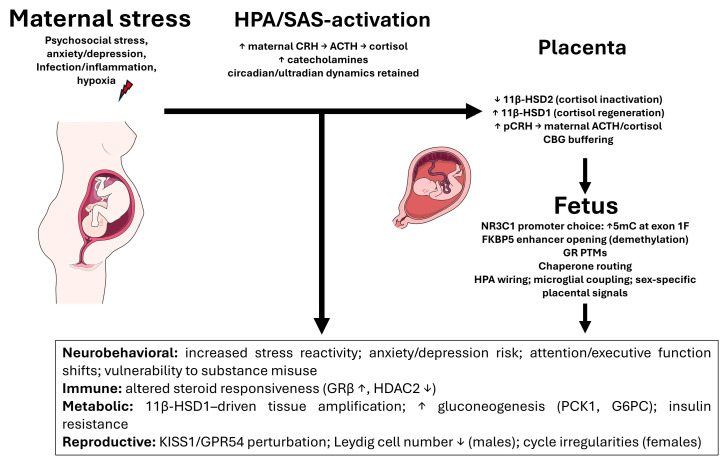
The placental modulation of maternal stress signals drives the fetal GR reprogramming. The resulting increase in active glucocorticoids reaching the fetus programs regulatory nodes: selective methylation across *nr3c1* first-exon promoters (e.g., ↑ 5mC at exon 1F), allele-specific opening of FKBP5 GRE enhancers, altered GR post-translational modifications and chaperone routing, and sex-biased signals that influence the HPA wiring and microglia coupling. The downstream phenotypes include: neurobehavioral—heightened stress reactivity, anxiety/depression risk, attention/executive shifts, vulnerability to substance misuse; immune–reduced steroid responsiveness (↑ GR, ↓ HDAC2), greater wheeze/asthma susceptibility, metabolic tissue “amplification” via 11β-HSD1, increased gluconeogenesis (PCK1, G6PC), insulin resistance; reproductive–disrupted KISS1/GPR54, fewer Leydig cells (males), and fewer cycle irregularities (females). The figure was created using Servier Medical Art (https://smart.servier.com/), licensed under CC BY 4.0 (https://creativecommons.org/licenses/by/4.0/ (accessed on 10 August 2025)).

Prenatal stress does not merely “raise cortisol”; it reconfigures the interlinked circuits of the maternal HPA and sympathoadrenal systems, the placental endocrine hub, the enzymatic barriers, and the epigenetic mechanisms, such that the fetus acquires a different baseline calibration of the stress, metabolic, immune, and reproductive axes (Figure 6). This calibration persists for years, is modulated by sex and environment, and can partly transmit to the next generation. These outcomes arise from complex interactions among molecular, cellular, and system-level mechanisms, including activation of the glucocorticoid system due to failed containment, oxidative stress, and epigenetic changes. Understanding these mechanisms is essential to developing effective strategies for the diagnosis, prevention, and treatment of non-genetic neurological, endocrine, metabolic, and autoinflammatory diseases, ultimately improving the quality of life for future generations.

## 7. Long-Term Consequences of Epigenetic Reprogramming of the GR in the Offspring

### 7.1. Stress-Associated Disorders

A classical rat study showed that the quality of maternal care reprograms *nr3c1* promoter methylation in the offspring hippocampus, reduces the GR expression, and heightens stress reactivity; these effects are reversible with HDAC inhibitors, pointing to a causal role for epigenetics [3]. This is the “canonical” mechanistic chain: ↑ DNA methylation in the 5′ regulatory region of *nr3c1* → ↓ the GR in the hippocampus → weakened the HPA negative feedback [332]. A similar pattern has been found in humans: hypermethylation of the *nr3c1* promoter (exon 1F) and reduced GR expression in the hippocampus of individuals who died by suicide and had a history of childhood trauma. The picture is complemented by FKBP5: in carriers of the risk allele [333], childhood trauma induces the allele-specific demethylation of GRE enhancers within FKBP5, rendering the GR complex less responsive and durably increasing stress reactivity in neural and immune systems. At the level of fine-tuning in the brain, age/stress-induced increases in local glucocorticoid regeneration via 11β-HSD1 worsen cognitive performance; partial deficiency or pharmacologic inhibition of 11β-HSD1 improves memory in aged mice [334]. When taken together, these links are consistent with the elevated risk of depression and PTSD following early adversity.

### 7.2. Vulnerability to Psychoactive Substance Misuse

The elevated basal HPA reactivity, together with a weakened GR brake, reshapes dopaminergic reward circuits [335]. In prenatal stress models, adult male rats show an enhanced self-stimulation by psychostimulants (amphetamine/cocaine) [335,336] and an altered population activity of VTA neurons [337]; the behavioral effects co-occur with an “epigenetic scar” across the GR/FKBP5 circuit similar to that of early-stress paradigms [338]. This points to a mechanism of increased salience of stress-relieving stimuli and sensitization of mesolimbic pathways under attenuated GR control.

### 7.3. Cardiometabolic Risk: Obesity, Insulin Resistance, Hypertension

The tissue amplification of glucocorticoids via 11β-HSD1 is metabolically consequential. The transgenic overexpression of 11β-HSD1 in adipose tissue induces visceral obesity [339], insulin resistance, and dyslipidemia; conversely, 11β-HSD1 protects mice from diet-induced insulin resistance and hyperglycemia. In the vascular system, deficiency of 11β-HSD2 (the placental “filter” during embryogenesis) and/or chronic glucocorticoid exposure programs renal and vascular regulation: preclinical models report heightened sympatho-adrenal activity, enhanced vasoconstriction, reduced baroreflex sensitivity, and the development of hypertension in adult animals exposed to prenatal stress/glucocorticoids [340]. The multifactorial arc is as follows: ↓ placental HSD11B2 and/or ↑ HSD11B1 → ↑ fetal glucocorticoid exposure → epigenetic remodeling of the HPA/RAAS/sympathotonic axes → vascular stiffening and a hypertensive phenotype [341].

### 7.4. The Reproductive Axis and Sex Differences

The sex-dependent mechanisms represent a critical dimension of prenatal stress biology [342]. The human evidence suggests that the placental handling of glucocorticoids, including 11β-HSD2/11β-HSD1 expression, the placental GR isoform composition, and the GR-linked signaling, as well as downstream HPA axis outcomes, can differ between male and female offspring [25,147,343,344]. A major source of this dimorphism is the placenta itself, which exhibits sex-specific adaptive strategies in response to adverse maternal conditions. Male placentas are often described as less plastic and more vulnerable to sustained intrauterine stress, whereas female placentas are more frequently engaged in compensatory responses that may ensure fetal survival at the cost of an altered developmental programming [25,343]. In human studies, sex-dependent differences have been reported in placental glucocorticoid metabolism, GR expression/localization, and FKBP51-related signaling, indicating that fetal sex influences not only the level of glucocorticoid exposure but also the intracellular interpretation of glucocorticoid signals at the fetoplacental unit [147,343,344].

These placental and molecular differences are reflected in sex-specific programming of the HPA axis and later behavioral phenotypes. The human evidence synthesized in systematic-review form suggests an increased vulnerability of the female HPA axis to prenatal programming, particularly with respect to stress reactivity [343]. The experimental data further support this conclusion. The recent work demonstrated that transgenerational maternal stress can produce sex-dependent biobehavioral phenotypes in the F3 generation, with females showing greater neuroimmune and behavioral sensitivity, including female-specific increases in IL-1β and IL-10, whereas males exhibited a phenotype more strongly associated with HPA axis hyperactivity [342]. In addition, in a rat model of prenatal stress, adolescent females, but not males, developed a clear depressive-like phenotype, while recognition–memory deficits were observed in both sexes; these changes were accompanied by oxidative stress-related alterations in the hippocampus and sex-dependent differences in TGF-β1-associated signaling [345]. Together with broader evidence on sex differences in stress biology, these findings indicate that fetal sex is a fundamental determinant of how prenatal stress is translated into long-term neuroendocrine and affective outcomes [327,342,345,346].

Beyond the HPA axis outcomes, prenatal stress can also recalibrate reproductive neuroendocrine pathways. GR-dependent fetal programming affects the hypothalamic–pituitary–gonadal (HPG) axis during critical windows of sexual differentiation and gonadal development [327,346]. In experimental models, prenatal stress reduces KISS1/GPR54 signaling in the hypothalamus, disrupts gonadotropin secretion, and impairs reproductive function in male offspring [347]. Other studies show that prenatal stress can also compromise fertility in both sexes, being associated with reduced testosterone levels, increased testicular cell death, and impaired sperm quality in males, as well as longer estrous cycles, reduced estradiol/progesterone, and fewer mature follicles in females [24]. These reproductive effects are mechanistically consistent with sex-specific placental glucocorticoid signaling and with the broader concept that prenatal stress perturbs interactions between glucocorticoids and gonadal steroids during developmental windows when neuroendocrine circuits are being organized [327,346]. Thus, fetal sex should be considered not simply as a modifying variable but as a fundamental determinant of how prenatal stress is encoded into behavioral, metabolic, immune, and reproductive trajectories later in life.

## 8. Conclusions

Glucocorticoid signaling in pregnancy emerges as a pivotal nexus between the maternal environment and fetal development. This review highlights how a transient maternal stress signal can be converted into a durable physiological imprint in the offspring. Specificity in glucocorticoid action arises not from “GR alone”, but from an entire regulatory ensemble: receptor isoforms, chaperone machinery, co-regulators, and chromatin context all determine the outcome of a given cortisol pulse. Crucially, the placenta functions as an active interpreter of maternal stress, buffering or amplifying the glucocorticoid transfer to the fetus in a context-dependent manner. When maternal stress, inflammation, or hypoxia overwhelms placental defenses (for example, by downregulating 11β-HSD2 and/or upregulating 11β-HSD1), the ensuing fetal glucocorticoid excess is etched into the epigenome. This leaves the fetus with a recalibrated HPA axis set-point and an altered developmental trajectory, manifesting as long-term differences in stress reactivity, cognitive-affective outcomes, metabolic and immune homeostasis, and reproductive function. The direction and magnitude of these changes depend on modifiers like fetal sex and timing (gestational stage and even time of day), which helps explain the heterogeneity of outcomes observed across individuals and cohorts.

From these insights, several clinical implications and opportunities for intervention can be outlined. Upstream, it is evident that minimizing maternal stress and inflammation during pregnancy is critical. Stress-reduction strategies, psychosocial support, and prompt treatment of infections or inflammatory conditions in expectant mothers could help maintain the placental barrier and normative fetal development. Ensuring robust circadian regulation (e.g., promoting healthy maternal sleep patterns and timing of light exposure) is another important consideration, since circadian misalignment may exacerbate the HPA axis disturbances. Clinicians should also exercise caution and precision with any necessary glucocorticoid therapies during pregnancy. While antenatal glucocorticoids (e.g., for fetal lung maturation) can be lifesaving, their timing and dosing should be optimized to achieve benefit while avoiding unnecessary fetal glucocorticoid exposure. In short, maternal stress management, inflammation control, circadian hygiene, and judicious use of glucocorticoids form the first line of defense in protecting the fetus.

At the placental and fetal tissue level, several potential intervention points have been identified. Sustaining or enhancing placental 11β-HSD2 activity (or preventing its stress-induced decline) could strengthen the metabolic barrier that shields the fetus from cortisol [269,281,282,348]. Conversely, selective inhibition of 11β-HSD1 in the placenta or fetal tissues might reduce the local regeneration of active glucocorticoids [91,293,294], thus dampening tissue-level overexposure. Downstream, in the offspring’s organs and cells, targeting key molecular players of the stress response is a promising approach. For instance, restoring the HDAC2 function in immune cells has been suggested as a way to improve the GR-mediated anti-inflammatory responses, since low HDAC2 activity is linked to inflammatory gene escape and steroid insensitivity [185,349]. Similarly, curbing the expression or effects of the GRβ isoform—a dominant-negative variant that can blunt GRα action—may help reverse glucocorticoid resistance and is an area of active research [64]. Another emerging avenue is the modulation of microRNA networks that are dysregulated by prenatal stress. Changes in specific microRNAs (including those originating from the placenta) have been implicated in shaping GR signaling and developmental outcomes, raising the possibility that microRNA-based therapies or biomarkers could be utilized to reset or monitor these pathways.

Looking forward, one of the most exciting implications of this work is the potential to use epigenetic markers as tools for risk screening and early intervention in obstetric practice. The same molecular changes that mediate fetal programming could serve as sentinels of elevated risk. For example, a pattern of increased DNA methylation at specific *nr3c1* promoters or an aberrant profile of placental microRNAs (detectable in maternal circulation) might flag pregnancies in which the fetus has experienced high stress exposure. Incorporating such biomarkers into prenatal care could enable the early identification of mother–infant dyads at risk for adverse outcomes, prompting timely supportive interventions (lifestyle, nutritional, or pharmacological) to mitigate long-term harm [256,257,258,261,262,263,264]. In the future, an epigenetic risk panel—assessing the key DNA methylation and miRNA indicators of fetal stress—might become part of the routine screening for vulnerable pregnancies, much like other prenatal tests for developmental disorders.

In sum, the interplay between maternal stress signals, placental regulation, and fetal epigenetic remodeling provides a compelling explanation for how brief prenatal perturbations can lead to long-lasting alterations in offspring physiology—including endocrine, immune, metabolic, and reproductive functions. Recognizing the modular nature of this mother–placenta–fetus network moves the field beyond merely observing associations and towards targeted interventions that can break the chain of adversity. By safeguarding the dynamic but delicate balance of the maternal–fetal stress axis (while preserving its necessary flexibility), we can strive to improve developmental and reproductive health outcomes for the next generation and even attenuate the echo of stress across generations.

## Figures and Tables

**Table 1:** The GR isoforms and physiological function.

GR Isoforms	Tissue Where Expressed	Physiological Function/Features	Sources
GRα-A (full length, 777 a/a)	In all tissues and in approximately equal amounts	Classic form	[114,115]
GRα-B (~752 a/a)	Liver, pancreas, stomach	Common transcriptional activity	[114,115]
GRα-C1/C2/C3 (~727–730 a/a)	Pancreas, colon, lungs	Increased gene activation, which is associated with the increased induction of apoptosis	[115,116,117]
GRα-D1/D2/D3 (~612–615 a/a)	Spleen, bladder, dendritic cells	Reduced transcriptional activity	[115,116,118]

**Table 2:** The major GR phosphorylation sites.

Phosphorylation Site	Enzymes	Effect on GR	Physiological Role	Sources
Ser203	CDK5, p38, MAPK	Decreases transcriptional activity stimulates accumulation in the cytoplasm	The regulation of receptor activity in response to the cell cycle and stress	[152,156]
Ser211	CDK2, p38, MAPK	Increases transcriptional activity and stimulates nuclear translocation	The main marker of activated GR is important for apoptosis and immune response	[142,152,153]
Ser226	JNK	Decreases transcriptional activity and accelerates export from the nucleus	Fine-tuning the response to stress and regulating the intensity of the hormonal signal	[41,42]
Ser134 *	p38, MAPK	Modulates interaction with 14-3-3ζ, reducing activation of several genes	The integration of stress signals and their impact on specific genetic responses	[157]
Ser404	GSK-3β	Suppresses transcriptional activity and affects gene activation profile	The regulation of the inflammatory response and control of interactions with co-activators	[158]

* In contrast to most phosphorylations, it occurs ligand-independently.

## Data Availability

No new data were created or analyzed in this study. Data sharing is not applicable to this article.

## Abbreviations

11β-HSD1/2	11β-hydroxysteroid dehydrogenase type 1/2
ACTH	Adrenocorticotropic hormone
AF-1	Activation function-1 region
CBG	Corticosteroid-binding globulin (transcortin)
CRH	Corticotropin-releasing hormone
CS	Corticosterone
DBD	DNA-binding domain
FKBP51/52	FK506 binding protein 51/52
GR (NR3C1)	Glucocorticoid receptor (nuclear receptor subfamily 3, group C, member 1)
GRE	Glucocorticoid response element
HPA	Hypothalamic–pituitary–adrenal axis
LBD	Ligand-binding domain
MR	Mineralocorticoid receptor
NTD	N-terminal domain
POMC	Pro-opiomelanocortin
PTMs	Post-translational modifications
